# Pertussis (Keuchhusten)

**DOI:** 10.1007/s10405-020-00345-2

**Published:** 2020-10-06

**Authors:** Ulrich Heininger

**Affiliations:** 1 Universitäts-Kinderspital beider Basel, Spitalstr. 33, 4056 Basel, Schweiz; 2 Medizinische Fakultät, Universität Basel, Basel, Schweiz

**Keywords:** *Bordetella pertussis*, Übertragung, Polymerase-Kettenreaktion, Antibiotika, Impfprävention, *Bordetella pertussis*, Transmission, Polymerase chain reaction, Antibiotics, Immunization

## Abstract

Pertussis wird durch das gramnegative Bakterium *Bordetella pertussis* verursacht. Die Krankheitsmanifestationen reichen von unspezifischem Husten bis zu lebensbedrohlichen Verläufen mit Hyperleukozytose und respiratorischer Insuffizienz, v.a. bei jungen Säuglingen. Die Diagnose basiert auf klinischer Symptomatik und mikrobiologischen Nachweisverfahren. Die Therapie besteht aus Makrolidantibiotika; bei Apnoen kann Koffein versucht werden. Die Inzidenz beträgt 10–40 Fälle/100.000 Bevölkerung und Jahr, bei Säuglingen ist sie am höchsten (ca. 50), gefolgt von Jugendlichen (30–35). Mehr als 50 % der in den ersten 5 Lebensmonaten an Pertussis erkrankten Kinder werden hospitalisiert. Die Impfprävention umfasst Grundimmunisierung und regelmäßige Auffrischimpfungen mit azellulären Impfstoffen. Um schwere Verläufe bei jungen Säuglingen zu verhindern, ist die Impfung schwangerer Frauen am erfolgversprechendsten. Säuglinge geimpfter Mütter sollen zeitgerecht ab dem Alter von 2 Monaten für den Eigenschutz immunisiert werden.

## Abstract (en)

Pertussis is caused by the gram-negative bacterium *Bordetella pertussis*. The disease manifestations range from unspecific coughing to life-threatening courses with hyperleukocytosis and respiratory failure, especially in young infants. The diagnosis is based on clinical symptoms and microbiological detection methods. The treatment consists of macrolide antibiotics and caffeine can be used against apnea. The incidence is 10–40 cases/100,000 inhabitants per year and highest in infants (approximately 50), followed by adolescents (30–35). In the first 5 months of life more than 50% of all children with pertussis are hospitalized. Vaccination prevention consists of basic immunization and regular booster doses with acellular component vaccines. Vaccination of pregnant women is the most promising strategy to prevent severe pertussis in young infants. Infants of vaccinated mothers should still be immunized in a timely manner for their own protection from the age of 2 months onwards.

## Lernziele

Nach Lektüre dieses Beitrags

- können Sie die Bedeutung der Pertussis im Kindesalter gut beurteilen.
- ziehen Sie Schlüsse aus der gegenwärtigen epidemiologischen Situation.
- identifizieren Sie zuverlässig Patienten mit Pertussis und kennen die richtige Behandlung der Krankheit.
- wenden Sie die verfügbaren Prophylaxemaßnahmen, insbesondere Impfungen, richtig an und tragen dazu bei, dass möglichst wenige Säuglinge an Pertussis sterben.

## Einleitung

Pertussis ist trotz verfügbarer Impfstoffe eine **häufige Infektionskrankheit**

häufige Infektionskrankheit.

Dies hat verschiedene Gründe: suboptimale Wirksamkeit der Impfstoffe, zu späte und unvollständige Durchführung der empfohlenen Impfungen sowie kontinuierlich nachlassender Impfschutz [1]. Ohne vollständige Kenntnis der Epidemiologie, konsequente Diagnostik, Meldung aller Krankheitsfälle und Umsetzung der bestehenden Impfempfehlungen wird diese Krankheit weiterhin einen hohen Tribut fordern. Jeder Pädiater sollte deshalb die Merkmale der Krankheit und ihre Präventionsmöglichkeiten kennen.

### Fallbeispiel

Lea erkrankt im Alter von 4 Wochen plötzlich an einer Rhinitis und rezidivierenden Apnoen. Dabei wird sie zyanotisch und wirkt wie abwesend. Kurz vor der Geburt war Leas Mutter (28 Jahre alt, letzte Pertussisimpfung im Alter von 14 Jahren) zu Besuch bei einer Freundin, die seit 2 Wochen an einem anfallsartigen Husten litt. Zehn Tage nach der Geburt erkrankte sie dann selbst an einem hartnäckigen Husten, der immer noch besteht. Sie ist sehr besorgt und bringt Lea zu ihrer Kinderärztin. Diese untersucht das Kind, findet außer der Rhinitis keine besonderen Krankheitszeichen. Aufgrund der Anamnese äußert sie den Verdacht auf Pertussis, entnimmt Nasopharyngealsekret für eine Polymerase-Kettenreaktion (PCR) und weist Lea in die nächste Kinderklinik ein. Lea wird kontinuierlich pulsoxymetrisch überwacht und erhält eine antibiotische Therapie mit Azithromycin. Sie hat etwa alle 20–30 min stimulationsbedürftige Apnoen mit Bradykardie und Abfall der Sauerstoffsättigung. Im Blutbild besteht eine Leukozytose von 52.000/µl mit 82 % Lymphozyten. Am nächsten Tag übermittelt das Labor das Ergebnis der PCR: *B. pertussis* im Sekret nachgewiesen.

## Erreger

Pertussis wird durch das gramnegative Bakterium **Bordetella pertussis**

Bordetella pertussis

hervorgerufen. Ein geringer, regional und im zeitlichen Verlauf variabler Anteil keuchhustenähnlicher Krankheitsbilder wird durch* B.-parapertussis-* oder *B.-holmesii*-Bakterien verursacht [1]. Wichtigster Virulenzfaktor von *B. pertussis* ist das **Pertussistoxin**

Pertussistoxin

(PT). Es inhibiert G‑Proteine, wodurch auch die charakteristische Leukozytose (durch Lymphozytose) bedingt ist [2].

## Krankheitszeichen

### Manifestation und Verlauf

Nach einer Inkubationszeit von 7 bis 10 Tagen manifestiert sich die Krankheit sehr variabel: von leichtem, unspezifischen Husten über wochenlang anhaltende, **intermittierende Hustenattacken**

intermittierende Hustenattacken

(„Keuchhusten“) bis hin zu lebensbedrohlichen Verläufen mit Hyperleukozytose und respiratorischer Insuffizienz. Letale Verläufe werden fast ausschließlich im jungen Säuglingsalter beobachtet [1, 3].

### Besonderheiten im Säuglingsalter

Junge Säuglinge ohne von der Mutter passiv erworbene Immunität gegen *B. pertussis* erkranken bei Exposition besonders schwer. Statt intermittierenden Hustenattacken können **rezidivierende Apnoen**

rezidivierende Apnoen

mit Bradykardien im Vordergrund stehen, die eine stationäre Überwachung und ggf. intensivmedizinische Betreuung erforderlich machen [4]. Gleiches gilt für den Fall einer **leukämoiden Lymphozytose**

leukämoiden Lymphozytose

im peripheren Blutbild, die durch den Effekt des PT hervorgerufen wird. Dieses Toxin wird nur durch *B. pertussis* exprimiert. Die Werte können 30.000 Zellen/µl und mehr erreichen. Gefürchtete Folgen der Lymphozytose sind die Bildung von Lymphozytenaggregaten im Lungenkapillargebiet und eine konsekutiv auftretende **respiratorische Globalinsuffizienz**

respiratorische Globalinsuffizienz,

die tödlich verlaufen kann [5].

Bei Pertussis durch Infektion mit *B. pertussis* (sic!) im Säuglingsalter ist ein Differenzialblutbild zur Früherkennung einer bedrohlichen Lymphozytose unverzichtbar [6]. Säuglinge, die bereits eine oder mehrere Impfdosen erhalten haben, bilden dank den vorhandenen Anti-PT-Antikörpern kaum noch eine Lymphozytose aus.

#### Cave

Bei Pertussis durch *B.-pertussis*-Infektion im Säuglingsalter ist ein Differenzialblutbild zur Früherkennung einer bedrohlichen Lymphozytose unverzichtbar.

## Diagnose

Die Diagnose einer Pertussis stützt sich auf die **klinische Symptomatik**

klinische Symptomatik,

ergänzt durch mikrobiologische Nachweisverfahren wie PCR und/oder serologische Bestimmungen (Tab. 1; [7]). Das sensitivste Nachweisverfahren in den ersten 2 bis 3 Krankheitswochen ist der Nachweis des Erregers mithilfe der **Polymerase-Kettenreaktion**

Polymerase-Kettenreaktion

(PCR) im Nasopharyngealsekret. Fehlen die typischen Krankheitszeichen (Hustenattacken, anschließendes Erbrechen und inspiratorisches Juchzen), wird die Diagnose häufig übersehen [4].

**Table 1:** 

Hustendauer (Wochen)	Säuglinge	Ab dem Alter von 6 (bis 12) Monaten
<2	PCR aus Nasopharyngealsekret	PCR aus Nasopharyngealsekret
2–3		PCR aus Nasopharyngealsekret* oder* Spezifische Pertussistoxin-IgG-Antikörper im Serum
≥3 Wochen		Spezifische Pertussistoxin-IgG-Antikörper im Serum

*PCR* Polymerase-Kettenreaktion

### Merke

- *Bordetella pertussis* siedelt sich auf dem zilientragenden Epithel des Nasopharynx, nicht aber im vorderen Nasenabschnitt an.
- Das Sekret für den Nachweis von *B. pertussis* muss tief aus dem Nasopharynx gewonnen werden (Absaugen oder Abstrich).

Ab der 3. Krankheitswoche ist die **serologische Untersuchung**

serologische Untersuchung

diagnostisch erfolgversprechend. Dabei wird in einer Serumprobe der Anti-PT-IgG-Wert bestimmt. Voraussetzung für eine gute Aussagekraft ist, dass der Patient mindestens 6 (bis 12) Monate alt ist, somit keine interferierenden maternalen Antikörper mehr im Blut hat und dass in den letzten 12 Monaten keine Pertussisimpfung stattgefunden hat (die ebenfalls zu erhöhten Antikörpertitern führen kann). Sind diese Voraussetzungen erfüllt, gelten Werte von mindestens 50 EU/ml als starker Hinweis auf eine kürzlich stattgefundene *B.-pertussis*-Infektion [7].

### Cave

In folgenden Fällen ist keine zuverlässige serologische Diagnose einer Pertussis möglich:

- bei Säuglingen im Alter unter 6 Monaten (*beachte*: passiv übertragene maternale PT-IgG-Antikörper) sowie
- bei Patienten mit einer Pertussisimpfung in den letzten 12 Monaten (*beachte*: Induktion von eigenen PT-IgG-Antikörpern).

### Fallbeispiel

Die Kinderärztin hat richtig gehandelt und bei V. a. Pertussis Nasopharyngealsekret für eine pertussisspezifische PCR von Lea entnommen. Eine serologische Antikörperbestimmung wäre nicht hilfreich gewesen, da diese allenfalls transplazentar übertragene mütterliche IgG-Antikörper nachgewiesen hätte.

## Therapie

Verschiedene Antibiotika, insbesondere **Makrolide**

Makrolide,

sind gegen *B. pertussis* wirksam. Empfohlene Substanzen, Altersgruppen und Dosierungen sind in Tab. 2 zusammengefasst [8]. Der Effekt einer Antibiotikabehandlung auf den Krankheitsverlauf ist gering, es sei denn, dass die Behandlung in der katarrhalischen Frühphase der Krankheit einsetzt, was in der Praxis selten gelingt. Jedoch beendet die Antibiotikagabe zuverlässig die **Kontagiosität**

Kontagiosität

des Patienten. Im Säuglingsalter kann zwar *B. pertussis* über die antibiotische Behandlungsdauer hinaus mithilfe der PCR im Nasopharynx nachgewiesen werden, die Übertragung des Erregers scheint aber dennoch effizient verhindert zu werden [9].

**Table 2:** 

Antibiotikum	Patientenalter	Tagesdosis		Therapiedauer (Tage)
		Empfohlen	Maximal	
Azithromycin	Ab Geburt	10 mg/kgKG (in einer Dosis)	500 mg	5
Clarithromycin	Ab einem Monat	15 mg/kgKG (in 2 Dosen)	1 g	7
Erythromycinestolat	Ab einem Monat	40 mg/kgKG (in 2 Dosen)	2 g	14
Trimethoprim(TMP)-Sulfamethoxazol	Ab 2 Monaten	8 mg (TMP)/kgKG (in 2 Dosen)	320 mg	14

Supportive Behandlungsmaßnahmen umfassen häufige kleinere Mahlzeiten und das Vermeiden von Hustenanfälle auslösenden Trigger-Faktoren, wie z. B. Racheninspektionen. Der Nutzen von Antitussiva, Sedativa, Mukolytika, β‑sympathikomimetischen Substanzen, Kortikosteroiden und Antihistaminika ist fraglich und nicht durch kontrollierte Studien belegt [1]. Bei wiederholten Apnoen kann jedoch ein Therapieversuch mit **Koffein**

Koffein

erfolgen. Bei einem im Alter von 4 Monaten an Pertussis erkrankten ehemaligen Frühgeborenen der 27. Schwangerschaftswoche kam es nach einer Dosis Koffein (20 mg/kgKG i.v.) binnen 1 h zu keinen weiteren die Apnoen mehr [10]. Die Autoren vermuten, dass das Koffein ähnlich wie bei unreifen Frühgeborenen den Atemantrieb stimulierte und weitere Apnoen dadurch verhindert wurden. Zudem könnte Koffein einen positiven Einfluss auf den durch Leukozytenaggregate in den Pulmonalgefäßen hervorgerufenen pulmonalen Hypertonus haben.

### Merke

- Die antibiotische Behandlung einer nachgewiesenen Pertussis zielt in erster Linie auf die Beendigung der Kontagiosität des Patienten.
- Der antibiotische Effekt auf den Krankheitsverlauf ist gering.

Lebensbedrohliche Blutbildveränderungen erfordern die Durchführung von **Austauschtransfusionen**

Austauschtransfusionen

[11]. Bei respiratorischer Insuffizienz kann eine **extrakorporale Membranoxygenierung**

extrakorporale Membranoxygenierung

(ECMO) lebensrettend sein [12].

### Fallbeispiel

Auch am 2. Hospitalisierungstag zeigt Lea weiterhin mehrmals pro Stunde Apnoen mit Zyanose. In der wiederholten Untersuchung des Blutbilds hat die Leukozytenzahl auf 76.000/µl mit 87 % Lymphozyten zugenommen. Lea wird daraufhin intubiert, beatmet und erhält eine Austauschtransfusion. Anschließend beträgt der Leukozytenwert 27.000/µl mit 91 % Lymphozyten, und die Frequenz der Apnoen nimmt ab. Nach 2 Tagen kann sie extubiert werden und verbleibt noch 12 weitere Tage unter dem Monitoring von Atmung und Herzfrequenz in der Klinik. Nachdem sie dann für 28 h keine Apnoen mehr aufweist, wird sie nach 16 Tagen Aufenthalt entlassen.

## Epidemiologie

*Bordetella pertussis* ist ein ausschließlich humanpathogenes Bakterium und wird durch **Tröpfchen**

Tröpfchen

von Mensch zu Mensch übertragen. Die Inkubationszeit beträgt 7 bis 10 Tage (Variationsbreite: 5 bis 21 Tage). Junge Säuglinge erkranken an Pertussis, nachdem sie durch Personen aus ihrem engen Umfeld, meistens der eigenen Familie, infiziert wurden. In etwa der Hälfte der Fälle bleibt die Ansteckungsquelle unklar, was gezielte Prophylaxemaßnahmen erschwert [13].

### Fallbeispiel

Die Infektionskette, die zur lebensbedrohlichen Pertussis bei Lea führte, ist klassisch: Ihre hochschwangere Mutter steckte sich kurz vor der Geburt mit hoher Wahrscheinlichkeit bei einer Freundin an. Diese litt an einem anfallsartigen Husten, typisch für Pertussis, aber offenbar nicht als solche erkannt. Auch als Leas Mutter selbst Husten entwickelt, fühlt sie sich nicht sehr krank, sodass kein Arztbesuch stattfindet. Erst die Kinderärztin stellt die richtigen Fragen an die Mutter, äußert die korrekte Verdachtsdiagnose und deckt die Infektionskette auf.

Pertussis betrifft Personen jeden Lebensalters, aber mit unterschiedlicher Häufigkeit und longitudinalen Veränderungen. Seit März 2013 [14] ist „Keuchhusten“ in Deutschland meldepflichtig. Die Meldedaten sind über die **SurvStat-Datenbank**

SurvStat-Datenbank

des Robert Koch-Instituts (RKI) frei zugänglich und werden nach verschiedenen Falldefinitionen stratifiziert [15]. In den Publikationen des RKI werden die klinisch-labordiagnostisch oder klinisch-epidemiologisch bestätigten Fälle mitgeteilt.

Die aktuellen Auswertungen können, wie folgt, zusammengefasst werden.

### Jährliche Inzidenz.

**Figure 1:**
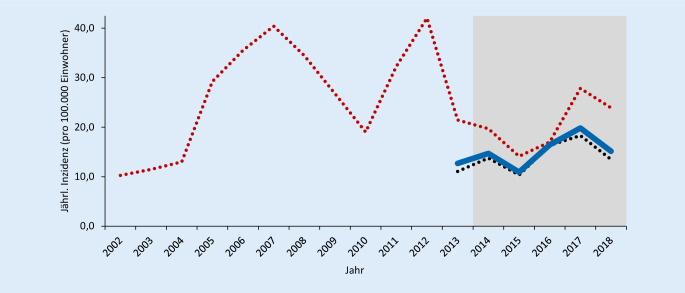

Die jährliche Inzidenz der an das RKI übermittelten Fälle variierte in den Jahren 2002–2018 unregelmäßig zwischen 10 und 40 Fällen/100.000 Einwohner. In Abb. 1 ist dies grafisch dargestellt. Vor Einführung einer bundesweiten Meldepflicht für Pertussis 2013 wurden nur Fälle aus den 5 östlichen Bundesländern Brandenburg, Mecklenburg-Vorpommern, Sachsen, Sachsen-Anhalt und Thüringen übermittelt (Abb. 1*rote gepunktete Linie*). Zur besseren Vergleichbarkeit mit der Periode vor der Einführung einer bundesweiten Meldepflicht sind ab 2013 die jährlichen Inzidenzen sowohl für alle 16 Bundesländer zusammen (Abb. 1*blaue Linie*) als auch unterteilt nach den 5 östlichen Bundesländern und den anderen 11 Bundesländern (Abb. 1*schwarze gepunktete Linie*) abgebildet. Zusätzliche Auswertungen wurden auf die bundesweiten Surveillance-Daten der letzten 5 Jahre beschränkt (2014–2018; Abb. 1*grauer Bereich*). Es zeigt sich, dass die Inzidenz derzeit in den westlichen Bundesländern niedriger ist als in den östlichen. Vermutlich liegt dies eher am Meldeverhalten, als dass es sich um einen echten Unterschied handelt.

### Altersabhängige jährliche Inzidenz.

**Figure 2:**
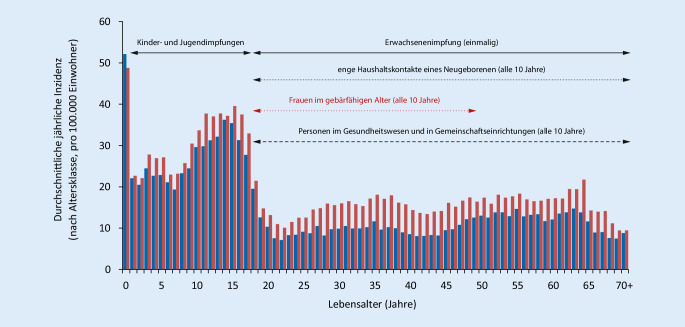

Im Zeitraum 2014–2018 hatten Säuglinge die **höchste altersabhängige jährliche Inzidenz der Pertussis**

höchste altersabhängige jährliche Inzidenz der Pertussis

in Deutschland (ca. 50/100.000), gefolgt von Jugendlichen im Alter von 10 bis 18 Jahren (30–35/100.000), Kindern im Alter von einem bis 9 Jahren (20–25/100.000) und Erwachsenen jeden Alters (10–20/100.000). In jeder Altersstufe, ausgenommen Säuglinge, ist die Inzidenz beim weiblichen Geschlecht höher als beim männlichen. In Abb. 2 sind neben der durchschnittlichen jährlichen Pertussisinzidenz (Fallzahlen nach Infektionsschutzgesetz, IfSG), differenziert nach Lebensalter und Geschlecht (*blaue Balken* männlich; *rote Balken* weiblich) im Zeitraum 2014–2018 zudem die derzeit gültigen STIKO-Impfempfehlungen für Pertussis ersichtlich (*durchgehender Pfeil* Standardimpfungen; *gepunkteter Pfeil* Indikationsimpfungen; *gestrichelter Pfeil* berufliche Indikationsimpfung; RKI 2019).

### Fallzahlen in den ersten 4 Lebensjahren.

**Figure 3:**
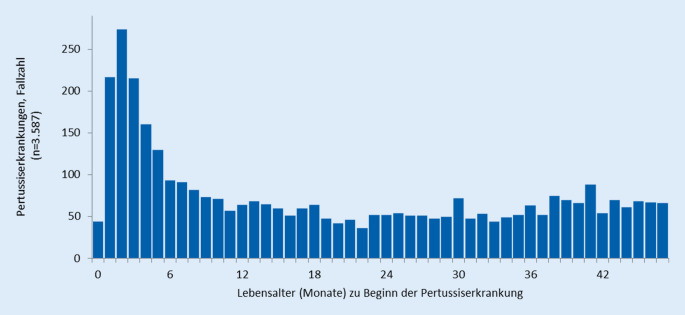

Bei genauer Betrachtung der Fallzahlen in den ersten 4 Lebensjahren ist der Gipfel im Alter von 2 bis 6 Monaten (Abb. 3) zu erkennen. Dies ist das Alter, in dem Säuglinge noch einen gewissen Nestschutz gegen viele andere Infektionskrankheiten aufweisen, von dem sie bis zum Erwerb des eigenen Schutzes durch Impfungen im Alter von 2 bis 4 Monaten profitieren. Diesen Krankheitsgipfel zu reduzieren, ist das Ziel der Pertussisimpfung bei Schwangeren (s. Abschn. „Impfprophylaxe“).

### Hospitalisierung.

**Figure 4:**
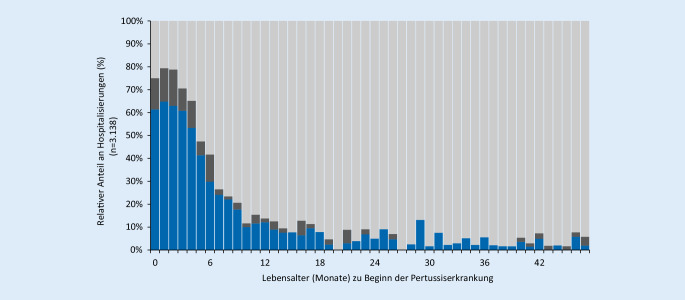

Mehr als 50 % der in den ersten 5 Lebensmonaten an Pertussis erkrankten Kinder werden hospitalisiert. Im übrigen Säuglingsalter geht der Anteil der Hospitalisierungen kontinuierlich auf etwa 10 % zurück und verbleibt bis zum Alter von 4 Jahren auf diesem niedrigen Niveau oder darunter (Abb. 4, Angaben zu Erkrankungsbeginn, Geburtsdatum und Krankenhausbehandlung wurden von 3138 Betroffenen im Alter <4 Jahre im Meldezeitraum 2014–2018 übermittelt; *blau* Hospitalisierung wegen einer Pertussis; *dunkelgrau* Hospitalisierungsgrund unbekannt oder Angabe eines anderen Hospitalisierungsgrundes bei gleichzeitig bestehender Pertussis; *hellgrau* nicht hospitalisiert)

### Merke

- Pertussis ist keine „Kinderkrankheit“, sondern betrifft Menschen jedes Alters.
- Am häufigsten und schwerwiegendsten verläuft die Pertussis im jungen Säuglingsalter.

## Meldepflicht, Isolationsmaßnahmen und Wiederzulassung in Gemeinschaftseinrichtungen

Für den Verdacht, die Erkrankung und den Tod an Keuchhusten besteht die **ärztliche Meldepflicht**

ärztliche Meldepflicht

gemäß §6 IfSG. Gemäß §7 IfSG besteht eine **Labormeldepflicht**

Labormeldepflicht

für den direkten oder indirekten Nachweis von *B. pertussis* und *B. parapertussis* [14].

Personen mit Pertussis sollen für 5 Tage nach Beginn der Antibiotikatherapie isoliert werden. Die **Wiederzulassung in Gemeinschaftseinrichtungen**

Wiederzulassung in Gemeinschaftseinrichtungen

nach Pertussis regelt §34 Abs. 1 IfSG, wie folgt:

- Für *Erkrankte* ist sie 5 Tage nach Beginn einer wirksamen Antibiotikatherapie (bei Gabe von Azithromycin nach 3 Tagen) oder 21 Tage nach Beginn des Hustens, wenn keine antibiotische Behandlung durchgeführt wurde, möglich.
- Für *Krankheitsverdächtige* ist die Wiederzulassung nach Vorliegen eines negativen Befunds mithilfe des Nukleinsäurenachweises (z. B. PCR) aus Nasopharyngealsekret möglich. Es sei denn, dass der behandelnde Arzt aufgrund der Gesamtbewertung aller vorliegenden klinischen und labordiagnostischen Befunde zu der Einschätzung kommt, der Patient könnte dennoch infektiös sein (falsch-negativer Befund). Im Übrigen gelten sinngemäß die Kriterien für die Erkrankten.

Als krankheitsverdächtig gelten gemäß IfSG Personen mit typischen Zeichen oder Symptomen einer Pertussis, sofern sie engen Kontakt zu einer Person mit bestätigter Pertussis während der Dauer der Ansteckungsfähigkeit hatten.

## Prävention

Pertussis kann durch postexpositionelle Antibiotikagabe und präexpositionelle Impfungen wirksam verhindert werden, wohingegen die Expositionsprophylaxe mangels spezifischer Frühsymptome infektiöser Kontaktpersonen insuffizient ist.

### Antibiotikaprophylaxe

Insbesondere Makrolidantibiotika sind gegen *B. pertussis* wirksam und werden zur Prophylaxe in gleicher Dosierung und Dauer wie bei der Therapie eingesetzt (Tab. 2).

Die **Postexpositionsprophylaxe**

Postexpositionsprophylaxe

mit einem Antibiotikum („Chemoprophylaxe“) wird von der STIKO [16] empfohlen für:

- „ungeimpfte Personen mit engen Kontakten zu einer erkrankten Person in Familie, Wohngemeinschaft oder einer Gemeinschaftseinrichtung“ sowie
- „geimpfte Personen mit engen Kontakten zu einer erkrankten Person, wenn sich in ihrer Umgebung gefährdete Personen (wie z. B. ungeimpfte oder nicht vollständig geimpfte Säuglinge, Kinder mit kardialen oder pulmonalen Grundleiden oder Schwangere im letzten Trimester) befinden“.

### Impfprophylaxe

In den meisten Ländern Europas sind ausschließlich sog. **azelluläre Pertussisimpfstoffe**

azelluläre Pertussisimpfstoffe

zugelassen, die ein bis 5 *B.-pertussis*-spezifische inaktivierte Antigene enthalten – alle zumindest das Pertussistoxoid. Sie gelten als vergleichbar effizient [1] und sind nur in Kombination mit Diphtherie- und Tetanustoxoid (und ggf. weiteren Komponenten wie inaktivierten Polioviren, *Haemophilus-influenzae*-Typ-b-Polysacchariden und „hepatitis B surface antigen“) verfügbar (Tab. 3). Pertussiseinzelimpfstoffe sind nicht erhältlich, wären aber z. B. für die Impfung von schwangeren Frauen wünschenswert [17].

**Table 3:** 

Impfstoff (Handelsname, Komponenten)	Hersteller bzw. Vertrieb	Pertussistoxoid	Filamentöses Hämagglutinin	Pertaktin	Fimbrienagglutinogene 2 und 3	Alterszulassung	Impfalter gemäß STIKO-Empfehlung^b^	Kommentare
Infanrix hexa (DTaP-HepB‑IPV+Hib)	GSK	25 µg	25 µg	8 µg	–	Säuglinge bis einschließlich Kleinkindalter^a^	2, 4 und 11 Monate	„Kleinkindalter“ ist nicht verbindlich definiert
Infanrix-IPV+Hib (DTaP-IPV+Hib)		25 µg	25 µg	8 µg	–	ab 2 Monate	2, 4 und 11 Monate	
Infanrix (DTaP)		25 µg	25 µg	8 µg	–	2–72 Monate	2, 4 und 11 Monate	
Hexyon (DTaP-HepB-IPV+Hib)	Sanofi Pasteur	25 µg	25 µg	–	–	6 Wochen–72 Monate	2, 4 und 11 Monate	–
Pentavac (DTaP-IPV+Hib)		25 µg	25 µg	–	–	2–24 Monate	2, 3, 4 und 11 Monate	–
Vaxelis (DTaP-HepB-IPV+Hib)	MSD	20 µg	20 µg	3 µg	5 µg	6 Wochen – einschließlich Kleinkindalter^a^	2, 4 und 11 Monate	„Kleinkindalter“ ist nicht verbindlich definiert
Boostrix (Tdap)	GSK	8 µg	8 µg	2,5 µg	–	≥48 Monate	5–6 Jahre, 9–17 Jahre, Erwachsene	Ab 40 Jahre zulassungskonform für Erstimmunisierung
Boostrix-Polio (Tdap-IPV)		8 µg	8 µg	2,5 µg	–	≥36 Monate	dito	dito
Covaxis (TdaP)	Sanofi Pasteur	2,5 µg	5 µg	3 µg	5 µg	≥48 Monate	5–6 Jahre, 9–17 Jahre, Erwachsene	Ab 12 Jahre zulassungskonform für Erstimmunisierung
Repevax (TdaP-IPV)		2,5 µg	5 µg	3 µg	5 µg	≥36 Monate	dito	dito
Tdap-IMMUN (Tdap)	Pfizer	25 µg	–	–	–	≥48 Monate	5–6 Jahre, 9–17 Jahre, Erwachsene	Ab 12 Jahre zulassungskonform für Erstimmunisierung

*STIKO* Ständige Impfkommission am Robert Koch-Institut

Pentavac ist nur für das „3+1“ Impfschema zugelassen (Stand 28.8.2020)

^a^Zur jeweils aktuellen Verfügbarkeit siehe www.pei.de → Arzneimittel → Impfstoffe → Lieferengpässe → Listen

^b^ Für Frühgeborene ist weiterhin das „3+1“ Impfschema mit einer zusätzlichen Dosis im Alter von 3 Monaten empfohlen.

#### Fallbeispiel

Im Entlassungsbrief von Lea werden keine Angaben zu den anstehenden Impfungen gemacht. Was tun? In Rücksprache mit einem Impfexperten erhält die Kinderärztin folgende Auskunft: Lea soll zeitgerecht im Alter von 2 Monaten die 1. Dosis DTaP-HepB-IPV+Hib erhalten.

Kinder, die an Pertussis erkrankten, entwickeln **keine dauerhafte Immunität**

keine dauerhafte Immunität

und sollten deshalb trotzdem gegen Pertussis geimpft werden. Zudem sind keine Impfstoffe ohne Pertussisantigene für die ab dem Alter von 2 Monaten empfohlene Impfprophylaxe verfügbar. Auch lässt die Anwendung der Pertussisimpfung in Gegenwart vorbestehender spezifischer Antikörper keine vermehrten Nebenwirkungen erwarten, sodass die komplette reguläre Impfserie bei zuvor an Pertussis erkrankten Kindern die einzig sinnvolle Maßnahme darstellt.

#### Cave

Säuglinge, die an Pertussis erkrankt waren, sollen dennoch pünktlich zu allen von der STIKO empfohlenen Zeitpunkten mit einem üblichen hexavalenten Impfstoff geimpft werden.

Nachdem in den 1950er-Jahren die ersten Pertussisimpfstoffe auf der Basis komplett abgetöteter *B.-pertussis*-Bakterien, **„Ganzkeimimpfstoffe“**

„Ganzkeimimpfstoffe“,

eingeführt wurden, kam es zum Rückgang der Krankheitslast. Diskussionen um angeblich durch die Pertussisimpfung verursachte Hirnschäden bei Säuglingen („Enzephalopathie“) führten in den 1970er-Jahren zum Aussetzen der Impfempfehlung in vielen Ländern, so auch in Deutschland. Im Nachhinein bestätigte sich, was viele Experten von Beginn an vermuteten: Die Behauptungen waren auf Koinzidenz und nicht auf Kausalität zurückzuführen [18]. Daraufhin wurde die Impfung in Deutschland rehabilitiert [19] und wird seit 1991 wieder empfohlen. Nichtsdestoweniger hatten die Diskussionen die Entwicklung der mutmaßlich sichereren, definitiv aber besser verträglichen oben genannten azellulären Vakzinen induziert und nach ausgedehnten klinischen Studien zu deren Zulassung geführt [1]. Seitdem haben die Pertussisimpfempfehlungen in Deutschland eine stetige Veränderung mit sukzessiver Erweiterung der Zielgruppen für den Impfschutz erfahren (Tab. 4). Der Hypothese, dass der in vielen Ländern beobachtete Wiederanstieg („reemergence“) von Pertussis auf den Wechsel von Ganzkeim- zu den weniger effizienten azellulären Impfstoffen zurückzuführen sei [20], kann nicht zugestimmt werden [21, 22].

**Table 4:** 

1991	Wiedereinführung als Standardimpfung mit 4 Impfdosen in den ersten beiden Lebensjahren (und Nachholimpfung für unzureichend geimpfte ältere Kinder)
Ab 1995	Zulassung und Einführung der ersten azellulären Pertussisimpfstoffe in Deutschland (monovalent, aP, und in Kombination mit Diphtherie- und Tetanustoxoid, DTaP)
1998	Wechsel von Pertussisganzkeim- hin zu azellulären Kombinationsimpfstoffen für alle 4 Dosen (1–3 im 1. Lebensjahr, 4 mit 11 bis 14 Monaten)
2000	Einführung einer 5. Dosis für Jugendliche im Alter von 9 bis 17 Jahren
2001	Erstmals Einführung einer Indikationsimpfempfehlung für Erwachsene: *Personal in Pädiatrie und Infektionsmedizin sowie in Gemeinschaftseinrichtungen für das Vorschulalter und Kinderheimen*
2003	Modifikation der Indikationsimpfempfehlung für Erwachsene: *Personal in Einrichtungen der Pädiatrie, der Schwangerenbetreuung und der Geburtshilfe sowie in Gemeinschaftseinrichtungen für das Vorschulalter und Kinderheimen*
2004	Einführung der „Cocoon“-Strategie durch Modifikation der Indikationsimpfempfehlung für Erwachsene durch eine Erweiterung auf *Frauen mit Kinderwunsch präkonzeptionell sowie bei anstehender Geburt für enge Haushaltskontaktpersonen (Eltern, Geschwister) und Betreuer (z.* *B. Tagesmütter, Babysitter, ggf. Großeltern), spätestens 4 Wochen vor Geburt des Kindes, sofern kein adäquater Immunschutz vorliegt*
	Definition des adäquaten Immunschutzes: *Impfung oder mikrobiologisch bestätigte Erkrankung innerhalb der vergangenen 10 Jahre*
2006	Einführung einer weiteren Standardimpfdosis für Kinder im Alter von 5 bis 6 Jahren (chronologisch die 5. Dosis, Beibehalten der Auffrischimpfung bei Jugendlichen als chronologisch 6. Standardimpfdosis). Indikationsimpfung für Erwachsene: da kein monovalenter Pertussisimpfstoff mehr verfügbar ist, wurde der Hinweis aufgenommen, *Tdap(-IPV) möglichst nicht früher als 5 Jahre nach der vorhergehenden Dosis der anderen im Impfstoff enthaltenen Antigene (Td) zu verabreichen*
	Weitere ergänzende Hinweise:
	*Im Zusammenhang mit erkannten Pertussishäufungen kann auch bei vollständig geimpften Kindern und Jugendlichen mit engem Kontakt zu Erkrankten im Haushalt oder in Gemeinschaftseinrichtungen eine Impfung erwogen werden, wenn die letzte Impfung länger als 5 Jahre zurückliegt*
	*Jede Auffrischimpfung mit Td (auch im Verletzungsfall) sollte Anlass sein, eine mögliche Indikation einer Pertussisimpfung zu überprüfen und ggf. einen Kombinationsimpfstoff (Tdap) einzusetzen*
2009	Einführung einer Pertussisstandardimpfung für alle Erwachsenen zum Zeitpunkt der nächsten fälligen Td-Impfung (einmalig als Tdap-, bei entsprechender Indikation als Tdap-IPV-Kombinationsimpfung)
	Modifikation der Indikationsimpfempfehlung für Erwachsene bei beruflicher Indikation: Für Personal in Gemeinschaftseinrichtungen wurde die Einschränkung „für das Vorschulalter“ aufgehoben sowie die Indikation einer Pertussisimpfung auf „Personal im Gesundheitsdienst“ (statt bisher „Personal in Einrichtungen der Pädiatrie, der Schwangerenbetreuung und der Geburtshilfe“) geändert. Der Zusatz *möglichst nicht früher als 5 Jahre nach der vorhergehenden Dosis der anderen im Impfstoff enthaltenen Antigene (Td)* wurde gestrichen und die Definition des adäquaten Impfschutzes (s. 2004) durch folgenden Hinweis ersetzt: *sofern in den letzten 10 Jahren keine Pertussisimpfung stattgefunden hat*
2010	Modifikation der Terminologie der Indikationsimpfempfehlung für Erwachsene (*Frauen im gebärfähigen Alter* statt Frauen mit Kinderwunsch)
2019	Überprüfung der Impfempfehlung für eine einmalige Pertussisimpfung im Erwachsenenalter: Die bestehende Standardimpfempfehlung eines *einmaligen Boosters für Erwachsene* soll zunächst beibehalten werden. Für Risikogruppen – a) Frauen im gebärfähigen Alter, b) enge Haushaltskontaktpersonen und Betreuende eines Neugeborenen und c) Personen, die im Gesundheitsdienst oder in einer Gemeinschaftseinrichtung arbeiten – bleibt es bei Auffrischimpfungen im Zehnjahresrhythmus
2020	Die Pertussisimpfung soll als Indikationsimpfung in jeder Schwangerschaft erfolgen
	Im Juni 2020 wurde das „2+1“ Impfschema mit Impfungen im Alter von 2, 4 und 11 Monaten eingeführt. Frühgeborene erhalten eine zusätzliche Dosis mit 3 Monaten.

Die Pertussisimpfung in der **Schwangerschaft**

Schwangerschaft

ist ein in vielen Ländern aktuell diskutiertes Thema [23], auch in Deutschland [24]. Sie gilt als sicher und effizient und sollte unabhängig vom mütterlichen Pertussisimpfstatus in jeder Schwangerschaft erneut erfolgen [25]. Ihr Ziel ist es – neben dem direkten Schutz der Schwangeren selbst – insbesondere das Neugeborene mit dem optimalen Repertoire an transplazentar übertragenen mütterlichen Antikörpern gegen *B. pertussis* auszustatten [26]. Dadurch können die bedrohlichen Krankheitsfälle bei jungen Säuglingen mit noch fehlendem oder unvollständigem eigenen Impfschutz wirkungsvoll verhindert werden. Als Folge der durch die mütterlichen Antikörper vermittelten **kindlichen Leihimmunität**

kindlichen Leihimmunität

könnte man vermuten, dass eine Verschiebung des 1. Impftermins vom Alter 2 auf z. B. 3 Monate sinnvoll wäre, um einen negativen Einfluss („blunting“) auf die eigene Immunitätsentwicklung beim Kind zu entgegnen [27]. Ein derartiges Vorgehen wird aktuell in den Niederlanden diskutiert [28]. Der Autor des vorliegenden Beitrags ist der Ansicht, dass dies wenig begründet ist. Zum einen kann ein negativer Effekt der zeitgerechten Impfung nicht konsistent gezeigt werden [29]. Zum anderen wird durch eine Verschiebung des Impfbeginns der Impfschutz gegen die anderen Krankheiten (wie z. B. invasive *Haemophilus-influenzae*-Typ-b[Hib]-Infektionen) verzögert, was ein unnötiges Risiko darstellt [30].

## Fazit für die Praxis

- Pertussis wird durch *Bordetella pertussis* verursacht, von Mensch zu Mensch übertragen und ist im jungen Säuglingsalter eine lebensbedrohliche Krankheit.
- Das empfohlene diagnostische Verfahren in den ersten 2 bis 3 Wochen seit Hustenbeginn ist der Erregernachweis mithilfe der Polymerase-Kettenreaktion (PCR) aus Nasopharyngealsekret, später dann serologische Bestimmungen.
- Der Effekt einer Antibiotikabehandlung (Makrolidantibiotikum) auf den Krankheitsverlauf ist gering, beendet aber zuverlässig die Kontagiosität des Patienten.
- Die Inzidenz beträgt aktuell in Deutschland ca. 10–40 Fälle/100.000 Einwohner; die höchste altersabhängige Inzidenz haben Säuglinge (im Mittel ca. 50/100.000 und Jahr).
- Kinder, die an Pertussis erkrankten, entwickeln keine dauerhafte Immunität und sollten deshalb trotzdem gegen Pertussis geimpft werden. Azelluläre Pertussisimpfstoffe gibt es nur in Kombination mit anderen Impfantigenen in variabler Zusammensetzung.
- Die Pertussisimpfung in der Schwangerschaft ist sicher und effizient. Die transplazentar übertragenen mütterlichen Antikörper schützen den jungen Säugling vor Pertussis.

## CME-Fragebogen

Ein 5 Wochen alter Säugling mit nachgewiesener Pertussis wird wegen Apnoen und Zyanoseanfällen in eine Kinderklinik eingewiesen. Welche der folgenden Untersuchungen ist essenziell?

Differenzialblutbild

Blutgasanalyse

Bestimmung des C‑reaktiven Proteins (CRP)

Ultraschall des Zentralnervensystems (ZNS)

Thoraxröntgen

Welches der folgenden Medikamente sollte bei einem Säugling mit häufig rezidivierenden Apnoen im Rahmen einer Pertussis als Therapie versucht werden?

Sedativum

Mukolytikum

β‑Sympathikomimetikum

Koffein

Kortikosteroid

Ein 5‑jähriges Kind hustet seit ca. 4 Wochen ohne Besserungstendenz. Sie vermuten eine Pertussis als Ursache. Welches Testverfahren ist am ehesten zur Diagnosesicherung geeignet?

Spezifische Pertussistoxin-IgM-Antikörper im Serum

Spezifische Pertussistoxin-IgG-Antikörper im Serum

Polymerase-Kettenreaktion (PCR) auf *B. pertussis* aus Nasopharyngealsekret

Polymerase-Kettenreaktion (PCR) auf *B. pertussis* aus Rachenabstrich

Blutbild und C‑reaktives Protein (CRP)

Welches ist das hauptsächliche Ziel der antibiotischen Therapie einer Pertussis?

Verkürzung der Dauer der Kontagiosität

Verkürzung der Hustendauer

Verhinderung einer sekundären Pneumonie

Behandlung der Leukozytose

Verhinderung von Hustenanfällen

In welcher Altersgruppe ist die altersabhängige Inzidenz der Pertussis in Deutschland am höchsten?

Jugendliche im Alter von 15 bis 17 Jahren

Schulkinder im Alter von 11 bis 14 Jahren

Schulkinder im Alter von 6 bis 10 Jahren

Säuglinge im Alter von 0 bis 5 Monaten

Säuglinge im Alter von 6 bis 11 Monaten

Für welche Situation besteht eine ärztliche Meldepflicht für Keuchhusten in Deutschland?

Verdacht, Erkrankung und Tod an Keuchhusten

Nur Erkrankung und Tod an Keuchhusten

Nur Erkrankung und Tod an Keuchhusten, sofern die Diagnose mikrobiologisch gesichert ist

Nur Tod an Keuchhusten

Nur Tod an Keuchhusten, sofern die Diagnose mikrobiologisch gesichert ist

Wie erfolgt die antibiotische Postexpositionsprophylaxe einer Pertussis bei einem 3 Wochen alten Neugeborenen?

Einmalgabe von Rifampicin

Gabe von 4 Dosen Rifampicin im 12-h-Intervall

Gabe von Azithromycin einmal täglich über 5 Tage

Gabe von Azithromycin einmal täglich über 3 Tage

Gabe von Clarithromycin 2‑mal täglich über 7 Tage

Wie viele Pertussisimpfdosen sind als Standardimpfungen nach aktuellem Stand der Empfehlungen (2020/2021) der Ständigen Impfkommission (STIKO) in Deutschland vorgesehen?

3

4

5

6

7

Wann sollte ein Kind, das aktuell im Alter von 4 Wochen an Pertussis erkrankte, gegen Pertussis geimpft werden?

Sofort

Sobald es wieder gesund und mindestens 2 Monate alt ist

Im Alter von 6 Monaten

Ab dem Alter von einem Jahr

Gar nicht, weil es natürliche Immunität erworben hat

Wann ist eine Pertussisimpfung in der Schwangerschaft sinnvoll?

Wenn die Schwangere in den letzten 10 Jahren keine Pertussisimpfung erhielt

Wenn die Schwangere in den letzten 5 Jahren keine Pertussisimpfung erhielt

Wenn die Frau das erste Mal schwanger ist

In jeder Schwangerschaft, unabhängig von Anzahl und Zeitpunkt früherer Pertussisimpfungen

Gar nicht, weil sie nur die Mutter vor Pertussis schützt, aber nicht das Neugeborene
